# Assessment of myocardial viability in low signal intensity areas on cine MRI comparison with late gadolinium enhancement imaging in patients with prior myocardial infarction

**DOI:** 10.1186/1532-429X-14-S1-P34

**Published:** 2012-02-01

**Authors:** Takashi Tanimoto, Keizo Kimura, Shingo Ota, Tomizo Masuno, Yuichi Ozaki, Kumiko Hirata, Takashi Kubo, Imanishi Toshio, Takashi Akasaka

**Affiliations:** 1 Cardiovascular Medicine, Wakayama Medical University, Wakayama City, Japan

## Background

Delayed-enhancement magnetic resonance imaging (DE-MRI) is highly effective for the diagnosis of myocardial infarction in both the acute and chronic phases, which is characterized as hyperenhancement after gadolinium injection. The use of gadolinium contrast, however, is limited in patients with advanced renal dysfunction and dialysis. On cine MRI, the signal intensity (SI) of non-infarcted area is equal to that of skeletal muscle and liver in normal subjects. It is well known that the SI of infarcted area is not uniform in patients with prior myocardial infarction. The significance of low SI in infarcted area on cine MRI has not been evaluated. The purpose of this study was to assess the myocardial viability of the area with low SI on cine MRI as compared with DE-MRI in patients with prior myocardial infarction.

## Methods

Fifty patients with prior myocardial infarction underwent both cine MRI and DE-MRI to assess the myocardial function and viability. The left ventricle was divided into 17 segments, resulted in a total of 850 segments. On cine MRI, the presence of low SI area and wall motion score (WMS) of each segments were assessed. On DE-MRI, the presence of late gadolinium enhancement (LGE) area, the thickness of LGE area and outer non-enhanced area, and the transmural extension of infarction were evaluated.

## Results

The low SI area on cine MRI was documented in 2.1±2.7 segments per patient and 105 of all 850 segments (12.4%). In DE-MRI, the LGE was detected in 6.6±3.7 segments per patient and 329 of all 850 segments (38.7%). The sensitivity, specificity, positive predictive value, negative predictive value, and accuracy to predict the myocardial necrosis by the presence of low SI area on cine MRI were 32%, 100%, 100%, 70% and 74%, respectively. Of 329 segments with LGE on DE-MRI, the segments with low SI showed thinner LGE area (3.0±1.0mm versus 3.3±1.3mm, p=0.037), thinner outer non-LGE area (1.1±1.0mm versus 3.3±2.0mm, p<0.001), greater transmural extent (76±14% versus 53±21%, p<0.001), and lower WMS (3.8±10.5 versus 1.5±0.8, p<0.001) than the segment without low SI.

## Conclusions

Although the sensitivity is relatively low, the presence of low SI area on cine MRI is a sigh of prior myocardial infarction. The area with low SI has poor viability.

## Funding

None.

